# The Use of Pit and Fissure Sealants—A Literature Review

**DOI:** 10.3390/dj5040034

**Published:** 2017-12-11

**Authors:** Reem Naaman, Azza A. El-Housseiny, Najlaa Alamoudi

**Affiliations:** 1 Pediatric Dentistry Department, Faculty of Dentistry, King Abdulaziz University, 21589 Jeddah, Saudi Arabia; dr.reem.naaman@hotmail.com or rnouman@stu.kau.edu.sa (R.N.); aalhosseiny@kau.edu.sa or ahussini@hotmail.com or azza.elhousseiny@dent.alex.edu.eg (A.A.E.-H.); 2 Pediatric Dentistry Department, Faculty of Dentistry, Alexandria University, 21526 Alexandria, Egypt

**Keywords:** pit and fissure sealants, caries prevention, resin-based sealants

## Abstract

This paper reviews the literature and discusses the latest updates on the use of pit and fissure sealants. It demonstrates the effectiveness of pit and fissure sealants in preventing caries and the management of early carious lesions. It compares the use of different sealant materials and their indications. It describes the application technique for sealants. It also reviews the cost-effectiveness of sealants as a preventive strategy. From this review and after the discussion of recently published studies on pit and fissure sealants, it is evident that sealants are effective in caries prevention and in preventing the progression of incipient lesions. It is therefore recommended that pit and fissure sealant be applied to high-caries-risk children for optimum cost-effectiveness. It is a highly sensitive technique that needs optimum isolation, cleaning of the tooth surface, etching, and the application of a thin bonding layer for maximum benefit. Recall and repair, when needed, are important to maximize the effectiveness of such sealant use.

## 1. Introduction

Dental caries is a multifactorial disease caused by alteration in the composition of the bacterial biofilm, leading to an imbalance between the demineralization and remineralization processes and manifested by the formation of caries lesions in primary and permanent dentitions [1]. The National Health and Nutrition Examination Survey (NHANES) 2011–2012 data showed that 37% of children, aged 2–8 years old, were diagnosed with dental caries in primary teeth, and 21% of children, aged 6–11, and 58% of children, aged 12–19, were diagnosed with dental caries in their permanent teeth. When comparing this data to the earlier survey of 1999–2004, an overall decline in the prevalence of caries in primary teeth and a slight decrease in the caries percentage in permanent teeth was noticed [2,3] (Table 1). However, this decrease was not found to be uniform across different age groups or consistent with sociodemographic status and different tooth surface sites. Instead, it was found that the greatest decrease in caries was among smooth surfaces rather than pits and fissures [4,5]. Pit and fissure caries accounts for about 90% of the caries of permanent posterior teeth and 44% of caries in the primary teeth in children and adolescents [6]. The use of caries preventive approaches, such as community water fluoridation, topical fluoride therapy, plaque control, and dietary sugar control, has been generally seen to be the cause of the overall decline of caries prevalence, which in turn has had a greater effect on smooth surface carious lesion reduction. The plaque retentive nature of pits and fissures make them difficult to clean, thereby causing them to be more susceptible to caries than smooth surfaces and possibly not to be protected by fluoride administration [7]. 

More effective measures are necessary to protect pits and fissures; these include the use of pit and fissure sealants. Sealant application is a preventive conservative approach involving the introduction of sealants into the pits and fissures of caries prone teeth; this sealant then bonds to the tooth micromechanically, providing a physical barrier that keeps bacteria away from their source of nutrients [8]. Despite the overall increases in sealant use, they are still considered to be underused worldwide although the efficacy and caries-preventive effect of pit and fissure sealants has been well documented in the literature.

Data from NHANES in 2011–2012, when compared to that from a previous survey in 1999–2004, showed an increase in the use of sealants in permanent teeth. About 31% of children, aged 6–8 years old, 49% of children, aged 9–11, and 43% of adolescents, aged 12–19, had at least one sealed permanent tooth [2,3,6] (Table 1).

In Europe, sealant prevalence in adolescents was found to be about 58.8% in Portugal and 8% in Greece [9,10]. In the Middle East, in Saudi Arabia, it was found that only 9% had a minimum of one permanent molar sealed [11].

The aim of this paper was to review the literature regarding the latest updates on the use of pit and fissure sealants on primary and permanent molars in children and adolescents. 

## 2. History of Fissure Sealant Development

In the past, several attempts were made to protect pits and fissures from becoming carious; approaches, such as enamel fissure eradication, were used. This involved the widening of the fissures, or so-called fissurotomy, to transform deep fissures into cleansable ones [12]. Another method was to treat pits and fissures with ammoniacal silver nitrate [13]. None of these approaches, however, had a great measure of success. A more invasive approach was introduced by Hyatt in 1923 and this involved the preparation of a class I cavity that included all deep pits and fissures and the placement of a prophylactic restoration. In fact, this approach remained the treatment of choice until the 1970s [14]. In 1955, Buonocore published his classic study, documenting the method of bonding of acrylic resin to previously etched dental enamel. He described the technique of acid etching, using 85% phosphoric acid for 30 s, as a tool to increase the adhesion of self-curing methyl methacrylate resin materials to dental enamel. This study was indeed the beginning of a revolution in dental clinical practice [15]. In the mid-1960s, Cueto generated the first sealant material, methyl cyanoacrylate, but it was not marketed. This material, however, was prone to bacterial disintegration in the oral cavity over time [16]. Later on, Bowen invented a viscous resin, called bisphenol-a-glycidyl dimethacrylate, and this has become known as BIS-GMA [17]. This class was found to be resistant to degradation and successfully produced a bond with etched enamel. Buonocore made further advances and published his first paper about pit and fissure sealant, describing his successful use of BIS-GMA resin with the use of ultraviolet light in 1970 [18]. 

## 3. Pit and Fissure Sealant Materials

**Figure 1:**
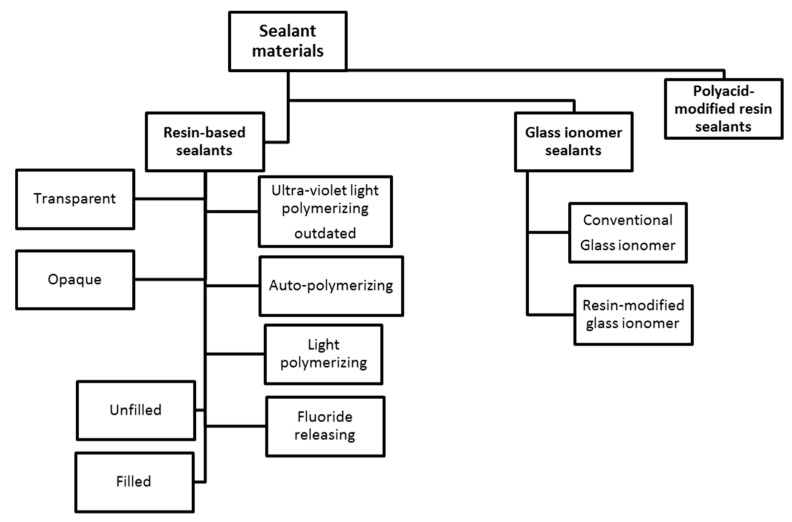
Classification of sealant materials.

Sealants are classified into three sealant materials (Figure 1). The predominant types of sealant materials in the market at present are resin-based sealants and glass ionomer cement-based sealants [19]. 

### 3.1. Resin-Based Sealant Materials (RBSs) 

Resin-based sealants (RBS) are classified into four generations, determined by the method of polymerization. The first generation of RBS was polymerized by the action of ultraviolet rays on the initiators in the material that initiate polymerization; this type, however, is no longer used [20]. Nuva-Seal^®^ (LD. Caulk Co.: Milford, DE, USA) was the sealant first introduced to the market and is an example of a resin-based sealant polymerized by an ultraviolet light source. The second generation was the auto-polymerizing resin-based sealants (ARBS) or chemically-cured sealants; tertiary amine (the activator) is added to one component and mixed with another component. The reaction between these two components produces free radicals that initiate the polymerization of the resin sealant material [20]. Such autopolymerizing resin-based sealants have now largely been replaced by the third generation, which comprises visible light-polymerizing resin-based sealants (LRBS). In this type of sealant, the visible light activates photoinitiators that are present in the sealant material and are sensitive to visible light in the wavelength region of around 470 nm (blue region) [21]. On comparing this visible light polymerizing to its previous generation, the autopolymerizing resin-based sealant, LRBS, sets in a shorter time, namely, 10–20 s, compared to the 1 to 2 min setting time of ARBS. The working time is longer and the material does not set until exposure to the polymerizing light. Through the elimination of the mixing step, fewer air bubbles are incorporated with the sealant application [22]. The fourth generation is the fluoride-releasing resin-based sealants (FRBS). Fluoride resin-based sealant is the product resulting from adding fluoride-releasing particles to LRBS in an attempt to inhibit caries. According to the literature, however, FRBS cannot be considered as a fluoride reservoir providing a long-term release of fluoride, and, as such, this kind of sealant provides no additional clinical benefit to LRBS [23,24,25]. 

RBS can also be classified according to their viscosity (filled and unfilled). The addition of filler particles to fissure sealant material seems to have only a small effect on clinical outcomes. Although filled sealants have a higher wear resistance, their ability to penetrate into fissures is low. The filled sealants usually require occlusal adjustments, which lengthen the procedure unnecessarily. The unfilled resin sealants on the other hand have a lower viscosity and provide greater penetration into fissures and better retention [23,26]. 

Sealant materials can also be classified according to their translucency (opaque and transparent) [23]. Opaque material can be white or tooth-colored, and transparent sealants can be clear, pink, or amber. White opaque fissure sealantsare easier to see during application and to detect clinically at recall examinations, compared to tooth-colored, opaque, or clear sealants [20]. A study has shown that the identification error was only 1% for the opaque resin sealant, compared to 23% for clear resin sealant [27]. However, the choice of the sealant material is usually a matter of personal preference.

Advances in the technology of resin sealant materials include the incorporation of a color change property. The change of this color property is either in the curing phase, such as Clinpro (3M ESPE, Saint Paul, MN, USA), or in the phase after polymerization, such as Helioseal Clear (Ivoclar Vivadent, Schaan, Liechtenstein). The advantage of this technology has not yet been fully proven but it may indeed offer the advantage of the better recognition of sealed surfaces [20,23]. It therefore seems that the most suitable choice of resin-based sealant would be the light polymerizing, unfilled, opaque sealant.

### 3.2. Glass Ionomer Sealant Materials 

Conventional glass ionomer (GI) material has also been used as pit and fissure sealants. It bonds chemically to enamel and dentin through an acid-base reaction between an aqueous-based polyacrylic acid solution and fluoroaluminosilicate glass powder [28]. GI sealants can be classified into low viscosity and high viscosity types. It is important to recognize that most of the studies on GI sealants used old-generation, low-viscosity GI, such as Fuji III GI sealant that has poor physical properties. It has now been replaced with a later generation, such as Fuji Triage (VII) (GC, Tokyo, Japan), that has better physical properties and is designed to release a higher amount of fluoride [29]. High viscosity glass ionomer cement (HVGIC), such as Ketac Molar Easymix (3M ESPE, Seefeld, Germany) and Fuji IX (GC, Tokyo, Japan), has been used in studies following atraumatic restorative treatment approach (ART). The ART concept consists of two components, namely, ART sealant and ART restoration. ART sealant is the preventive component that includes the application of HVGIC on vulnerable pits and fissures using the finger-press technique [30].

When resin is incorporated with glass ionomer, it is called a resin-modified glass ionomer (RMGI). It has also been used as a pit and fissure sealant material. The setting reaction of this type of sealant is initiated by the photoactivation of the resin component, followed by the acid-based reaction for the ionomer component. Its resin component has improved its physical characteristics, compared to conventional GI [22]. In fact, when compared to conventional GI, RMGI has less sensitivity to water and a longer working-time [28]. 

In general, the main advantage of a glass ionomer cement-based sealant is the continuous fluoride release and the fluoride recharging ability. Its preventive effect may even last after the visible loss of the sealant material as some parts of the sealant may remain deep in the fissures. It is moisture-friendly and easier to place and is not vulnerable to moisture, compared to the hydrophobic resin-based sealants [22]. It can be used as a transitional sealant when resin-based sealants cannot be used due to difficult moisture control in, for example, partially erupted permanent teeth, especially when the operculum is covering the distal part of the occlusal surface [31]. GI sealant can also be useful in deeply fissured, primary molars that are difficult to isolate due to a child’s pre-cooperative behavior [20]. It is considered a provisional sealant and has to be replaced with a resin-based sealant when better isolation is possible [32].

### 3.3. Polyacid-Modified Resin Based Sealants

Polyacid-modified, resin-based composite material, which is also referred to as compomer, has been used as a fissure sealant. It combines the advantageous properties of a visible light polymerized resin-based sealant with the fluoride releasing property of the GI sealant. A polyacid-modified resin-based sealant has a better adhesion property to enamel and dentin and is also less water-soluble, compared to GI sealant material [33], and less technique-sensitive, compared to resin-based sealants.

## 4. Different Sealant Materials and Caries Prevention

### 4.1. Sealant vs. No Sealant

The role of fissure sealants in caries prevention is well established in the literature. There is also a moderate quality of evidence that sealants reduce the incidence of caries by 76% on sound occlusal surfaces, compared to the non-use of sealants during the two to three year follow-up period [28].

A recent update of a Cochrane review evaluated the caries preventive effect of sealants in children and adolescents, compared with a no sealant control group. Thirty eight trials with a total of 7924 participants, aged between 5 and 16 years old, were included. Fifteen trials compared resin-based sealants when applied to the first permanent molars with no sealant and showed a moderate quality of evidence that resin sealants reduced caries increment by between 11 percent and 51 percent in a two year follow-up period. If caries increment was 40 percent in control teeth surfaces, the application of sealant reduced the caries increment to 6.25 percent. At longer follow-up periods of 48 to 54 months, the caries preventive effect of sealants was retained but the quality of evidence was low [34]. This is in agreement with the results of the previously published review [35].

When comparing the caries preventive effect of glass-ionomer based sealants with the use of no sealant, no conclusion could be drawn on whether GI sealant prevented caries, compared to no sealant, at a two year follow-up, due to the very low quality of evidence [34,36].

### 4.2. Sealant vs. Fluoride Varnish

Several published studies compared pit and fissure sealants’ effectiveness to that of fluoride varnish in caries prevention on occlusal surfaces. A recent update of a Cochrane review concluded that there is only a low quality of evidence that pit and fissure sealants have a superior outcome, when compared to fluoride varnish application, in the prevention of occlusal caries. This conclusion is similar to that found in the previous review in 2010 [37]. Two out of three studies included in the last updated review showed a significantly better performance of sealants, compared to fluoride varnish, while the third study reported that the benefits of sealant were not statistically significant, compared to fluoride varnish. Two of the included studies were assessed as having a high overall risk of bias and the third as having an unclear overall risk of bias [38]. The recent evidence-based guidelines of the American Dental Association (ADA), in collaboration with the American Academy of Pediatric Dentistry (AAPD), recommend the use of sealants in preference to no sealant or fluoride varnish, although the quality of evidence for this recommendation was found to be low [5,28]. In fact, this runs counter to the results from a recent randomized clinical trial that compared the clinical effectiveness for caries prevention of fluoride varnish and fissure sealants at a three year follow-up among a 6 to 7 years old population. After three years of follow-up, 17.5% of the fluoride varnish group and 19.6% of the fissure sealant group developed caries in their dentin. The difference between the two groups was not statistically significant [39]. 

### 4.3. Resin Based Sealant vs. Glass Ionomer Sealant

The caries preventive effect of resin-based sealants was compared to GI-based sealants in a recent update of a systematic review. Six trials were included in the meta-analysis and they found no statistical significant difference in the caries preventive effect when comparing RBS with GI-based sealants at 24, 36, and 48-month follow-up periods. At the 60-month follow-up, there was a border-line significance in favour of GI-based sealants. However, all the included trials were judged to be at a high risk of bias [40]. The outcome was therefore in agreement with the previously published review [41]. However, the recent update was more concerned with studies about HVGIC rather than low viscosity GIC sealants. 

A meta-analysis investigated the survival rates of atraumatic restorative treatment (ART) sealants and restorations using high viscosity glass ionomers (HVGIC). It was concluded that ART sealants have a high caries preventive effect (97%) after three years of application and a survival rate of 72%. This would make them effective alternatives to resin-based sealants [42].

Another recent clinical trial compared the effectiveness of caries prevention between GI sealants and RBS after 48 months and found four carious lesions in the GI group and 12 in the RBS group, but the difference was not statistically significant. The GI sealant was therefore shown to be an effective measure in caries prevention, although it had a significantly lower retention rate compared to RBS [43]. A possible reason behind the caries preventive effect of of GIC, despite it not being as retentive as RBS, is that GI remains in the deepest areas of the fissures, even though it is not clinically evident [44]. When HVGIC is used as part of the ART sealant technique, as described earlier, the sealant may penertrate even deeper into the fissures, compared to the conventional sealant application technique [40]. The anti-caries effect is also related to the fluoride release property of the cement [45]. Nevertheless,in a recent update, the American Dental Association recommendations, in collaboration with the American Academy of Pediatric Dentistry, could not draw any conclusion as to which of the two sealant materials was better due to the very low quality of the evidence available. Table 2 summarizes their findings from comparing the different sealant materials (Table 2) [5,28].

In partially erupted teeth, GI-based sealants may give better results in caries prevention effectiveness, compared to resin-based fissure sealants. A clinical trial compared the retention, marginal staining, and caries prevention properties of GI-based sealants and RBS, when placed on partially erupted, permanent molars. Thirty-nine molar pairs were included in the trial. Both types of sealants had no significant difference in their retention rates; however, the marginal staining was significantly higher for RBS. The GI-sealant group had no caries, while the RBS group had two carious molars and some others showed signs of demineralization. It was concluded that GI might be the better material for sealing partially erupted molars, as well as when salivary contamination is expected [31].

## 5. Sealant Retention of Different Materials

Previously, when investigating the effectiveness of sealants in preventing caries, half-mouth study designs were used, in which sealed teeth were compared with unsealed teeth as controls. Once the protective role of pit and fissure sealants was established in the 1980s, this type of study design became unethical. In other words, it was no longer acceptable to leave teeth with no sealant as a control after the efficiency of sealant in preventing caries had been proven. Since then, the retention rate has become the true determinant and a valid surrogate endpoint for sealant effectiveness in preventing caries [24,46]. The retention rate in most studies is classified into “intact sealant”, “partial loss”, and “complete loss” [47]. However, Mickenautsch and Yengopal in their recent systematic review do not support the use of sealant retention as a valid predictor for caries manifestation [48,49].

A systematic review evaluated the retention rate of the different materials of resin-based sealants (RBSs) placed on permanent molars. There was no significant difference between the complete retention of LRBS and ARBS. No statistically significant difference was observed when comparing LRBS with FRBS either at eight or 12 months. However, at the 48-month follow-up, the results indicated a significantly better retention for LRBS compared with FRBS. The overall decrease in the complete retention rate was observed over time in all types of sealant materials [24].

A recent meta-analysis investigated the clinical retention rates of pit and fissure sealants with regard to different types of materials at different observation-times. The resin-based sealants showed the best retention rates: the five-year retention rates for light-polymerizing, autopolymerizing, and fluoride-releasing resin-based sealants were 83.8%, 64.7%, and 69.9%, respectively. The GI-based fissure sealants, on the other hand, had a 5.2% retention rate at the five-year observation-time. Polyacid modified resin sealants also showed low retention rates [46]. However, studies that used HVGIC [50,51] or Fuji Triage [52] have shown improved retention rate results for GI sealants that are comparable to resin-based sealants.

When comparing filled and unfilled resin-based sealant retention rates, a study evaluated the retention of resin-based filled sealant Helioseal F (Ivoclar Vivadent, Schaan, Liechtenstein) and resin-based unfilled sealant Clinpro (3M ESPE, Saint Paul, MN, USA). They concluded that unfilled resin-based sealants showed slightly higher retention rates at the 12-month follow-up compared to those for filled resin-based sealants. Complete retention was 53.57% for filled RBS and 64.39% for unfilled RBS, but the difference was not statistically significant. Sealants without fillers appear to have better penetration into fissures than sealants incorporating filler particles, due to their lower viscosity [26].

The recent update of the American Dental Association’s recommendations, in collaboration with the American Academy of Pediatric Dentistry, reported that the GI sealant retention loss is five times greater compared to RBS and three times greater compared to RMGI sealant. The difference here is statistically significant, but the quality of evidence was assessed as being very low (Table 2) [5,28].

## 6. Techniques for the Placement of Resin-Based Sealants 

### 6.1. Tooth-Cleaning, Enamel Preparation, and Tooth Surface Treatment Prior to Sealant Placement

Most of the manufacturers’ instructions for the use of fissure sealants recommend careful cleaning of the pits and fissures before acid etching. A study reported that there is in fact no difference in sealant retention between toothbrush and handpiece prophylaxis at two to five year follow-ups [53]. 

Some manufacturers’ instructions state that the use of fluoride before sealant placement is contraindicated as it decreases enamel solubility in acid and thus inhibits proper etching of the enamel. However, Warren et al. compared the sealant retention of two sealant materials before and after fluoride treatment over an 18-month period. They reported a significantly greater retention on fluoridated teeth when LRBS was used and no significant difference in retention when ARBS was used. This suggested that sealant retention may not be impaired by fluoride application immediately prior to sealant placement [54]. Similarly, another study reported that the use of fluoride-containing prophylaxis paste or any fluoride treatment before sealant application does not adversely affect the sealant’s bonding to enamel [55]. One more study also evaluated sealant retention when treating the enamel with a topical fluoride gel before acid etching clinically and in-vitro. It was found that there was no statistically significant difference between the retention rate of the sealant applied after tooth surface treatment with topical fluoride and the control group that did not receive any fluoride treatment prior to the sealant application [56]. Furthermore, many studies have investigated different methods of mechanical preparation of the fissures, such as air abrasion, eliminating fissures with a dental bur, and sandblasting, prior to the sealant placement . Interestingly, it was found that fissure eradication is not necessary. Enameloplasty, using any of the above-mentioned techniques, removes the enamel layer overlying the dentin at the bottom of the fissure, making the tooth more susceptible to caries if the sealant is lost [20,57]. There is conflicting and limited evidence regarding the benefits of using a bur for fissure cleaning or for the purpose of increasing retention, prior to sealant placement [32,58].

### 6.2. Isolation

Adequate moisture isolation during resin sealant placement is the most critical step in sealant application. If the etched enamel gets exposed to salivary proteins for as little as 0.5 s, it can be contaminated [36]. If this occurs, re-etching is required. The use of a rubber dam is the ideal way to achieve optimum moisture control. The use of cotton rolls and a saliva ejector is also a valid option [59]. The use of moisture control systems, such as the Isolite^®^ system (Innerlite Incorporation, Santa Barbara, CA, USA) provides less time for the procedure and offers comparable sealant retention rates to cotton roll isolation or the use of a rubber dam [60].

A systematic review has suggested that four-handed delivery, compared to two-handed delivery, increases sealant retention by 9% when other factors, such as the surface cleaning method, were controlled [61]. The use of the four-handed technique facilitates sealant placement and is also associated with improved retention [32]. 

### 6.3. Acid Etching and Rinsing

The phosphoric acid concentration that was originally used for etching by Buonocore in 1955 was 85%, but it was then reduced in his early clinical studies to 50% [18]. Nowadays, 35% and 37% are the commonly used concentrations. Acid-etching times have also been reduced from 60 s down to 20 s [62].

Early recommendations for primary teeth enamel etching time were double the accepted time for permanent enamel, namely, 120 s for primary enamel and 60 s for permanent enamel. The early in vitro studies showed that 120 s are necessary for an adequate etching pattern in primary teeth enamel to eliminate the identification of prismless enamel. This finding was found not to be clinically significant for sealant retention, as demonstrated by Simonsen et al. in 1978. His study included 56 children between the ages of 3–8 years with 373 deciduous first and second molars that were sealed and examined six months post-application; 178 teeth were etched for 60 s and 195 teeth were etched for 120 s. The retention rate for the 60 s etched teeth was 100%, and for the 120 s etched teeth, it was 99% [8,23]. Moreover, the shorter etching time decreases the chance of saliva contamination, particularly in pre-cooperative children.

An in-vitro study evaluated the etching depth and bonding strength of 130 exfoliated primary teeth after the following four different etching times: 15, 30, 60, and 120 s. Despite the greater increase in depth after 120 s etching time, the mean bond strengths obtained for the four etching times were not significantly different [63]. Another study showed that the length of etching time has little effect on sealant retention. No significant difference in fissure sealants’ retention on primary or permanent molars was found after a one-year follow-up with different etching times of 15, 30, 45, and 60 s [64]. 

A rinsing time of 30 s and drying the tooth for 15 s should be sufficient to remove all acid etchant residues and achieve the characteristic chalky white enamel frosty appearance [20,22].

### 6.4. Bonding Agents

The idea of using a bonding agent under the sealant came from Feigal et al. in 1993 when they used hydrophilic bonding materials to aid the bond strength when the sealant is applied in a moist environment [65]. 

There have already been eight generations of bonding agents [66,67,68], the latest and eighth one being introduced in 2010. It is characterized by the incorporation of nano-fillers into the adhesive composition to improve the mechanical properties of the adhesive system. However, the most recent type in adhesive dentistry is called the universal adhesive or the multi-mode adhesive. It was first introduced in 2011. This kind of adhesive system can be used as an etch and rinse adhesive, a self-etch adhesive or to do self-etch on dentin and etch-and-rinse on enamel; this particular technique is called selective enamel etching. Its composition differs from the other adhesive systems that allow chemical and micromechanical bonding [67]. All the various adhesive types are summarized in Table 3. Several studies evaluated the use of a bonding agent before sealant application. A randomized controlled trial compared fourth generation (three-step-etch-and-rinse) and fifth generation (two-step-etch-and-rinse) adhesives when used under sealants. They found that the two-step adhesives reduced the risk of sealant loss by half (Hazard ratio = 0.53) when applied on occlusal surfaces. On the other hand, the three-step adhesives had a detrimental effect on the sealant retention rate, which can be explained by the composition of the adhesive, as it is water-based, and water has a deleterious effect on sealant bonding. The two-step adhesive is acetone- or ethanol-based, which may be more effective in bonding to etched enamel [69]. 

With regard to self-etch adhesives, a recent clinical trial evaluated the sealant retention rate and caries preventive efficacy over a three-year period. They compared three adhesive generations, namely, fourth generation (three-step-etch-and-rinse), fifth generation (two-step-etch-and-rinse), and sixth generation (one-step, two-component-self-etch) with the conventional technique, which is etching with no adhesive application as a control. There was a significant difference between the retention rates of sealants combined with the various adhesive systems used (*p* < 0.05). The highest retention rates of sealants on the first permanent molars at a 36-month recall were combined with the fourth and fifth generation adhesive systems and were 80.01% and 74.27%, respectively. In contrast, the lowest retention rates were combined with the sixth generation adhesive system (42.84%) and with the conventional acid-etch technique (62.86%). They also found that the fissure caries incidence rate in first permanent molars that had been sealed after using the sixth generation adhesive system was 34.28%, which was significantly higher than when other adhesive systems had been used [70]. This was in agreement with a previously published study that reported a significantly better retention rate with the etch-and-rinse adhesive system (fifth generation) compared to the self-etch adhesive system (sixth generation) at a 12-month follow-up [71]. Another study, on the other hand, evaluated the retention rate of fissure sealants in primary molars using a sixth generation (one-step, two-component-self-etch) adhesive compared to the conventional phosphoric acid-etching technique with no bonding agent application. They found no statistically significant difference in sealant retention in the two groups after a one-year follow-up period [72].

A recent systematic review compared the retention rate of sealants, combined with self-etch adhesive systems(sixth or seventh generation), with that of etch-and-rinse adhesive systems (fourth and fifth generations). Five studies were involved: three studies showed that etch-and-rinse adhesive systems had significantly better retention than self-etch adhesive systems. The other two included studies showed no significant difference between the two adhesive systems. Feigal and Quelhas in 2003, for example, reported similar retention rates of 61% at 24 months. However, the sample in this study was small (18 molars only) [73]. The systematic review concluded that the retention of occlusal fissure sealants is higher when applied with the etch-and-rinse adhesive system than with the self-etch adhesive system [74]. 

Finally, a recent systematic review by Bagherian et al. evaluated the fissure sealant retention rate with or without the use of an adhesive system and also compared the retention rate of sealants when using etch-and-rinse adhesive systems (fourth or fifth generations) versus the rate achieved when self-etching adhesive systems (sixth or seventh generations) were used. They found that the adhesive system has a positive effect on the retention of the fissure sealant. The adhesive components may increase the penetration into enamel porosities and thus increase bond strength. It was also found that etch-and-rinse adhesive systems are superior to self-etch adhesive systems in terms of sealant retention [75]. However, in a recent, randomized controlled trial, Khare et al. evaluated the integrity of fissure sealants by comparing the use of fifth, seventh, or Universal bonding systems with a no bonding protocol at 3-, 6- and 12-month follow-ups. At the 12-month follow-up, fifth generation bonding and universal bonding protocols performed better than seventh generation or no-bonding protocols, but the difference between the groups was not statistically significant [76].

In summary, the above-mentioned studies indicated that the use of adhesive systems prior to fissure sealant application had a positive effect on increasing penetration and improving the retention rate. It also appears that the use of bonding-agents that involve a separate acid-etching step (fourth and fifth generations) provides better sealant retention than self-etching adhesives (sixth and seventh generations). Etch- and-rinse adhesive systems produce better penetration of the enamel surface than self-etch adhesive systems, and this may result in a better bond strength.

An evidence-based 2008 report from the American Dental Association and the American Academy of Pediatric Dentistry supports the use of adhesive systems before sealant application for better sealant retention [32,58]. 

### 6.5. Sealant Evaluation After Placement

After curing the sealant and before the removal of the isolation material, the operator should examine the sealant for any voids, bubbles, or deficient material. Sealant retention should also be checked using the explorer in attempt to remove the sealant. If the sealant is dislodged, the fissures should be re-checked for any remaining food debris that may have caused the debonding of the sealant material. The tooth should be re-etched and a new sealant material should be applied. The operator should also be cautious enough to remove excess sealant material over the distal margin that may create a ledge [44].

## 7. Sealing Primary Teeth 

On the basis of caries risk assessment, primary teeth can be judged to be at risk due to fissure anatomy or patient caries risk factors, and would therefore benefit from sealant application [55]. Therefore, pit and fissure sealants are indicated in primary teeth, if such teeth have deep retentive or stained pits and fissures with signs of decalcification or if the child has caries or restorations in the contralateral primary molar or any other primary teeth [44]. Sealing should be considered particularly for children and young people with medical, physical, or intellectual impairment [59].

Pit-and-fissure sealants were found to be retained on primary molars at a rate of 74 to 96.3% at one year and 70.6–76.5% at 2.8 years [58]. However, the focus of most sealant studies is the occlusal surfaces of permanent molars and there is still insufficient evidence to support the use of fissure sealants in primary molars [32]. Rathnam and Madan maintain that it is difficult to conduct clinical studies on primary teeth due to several confounding factors, such as age, cooperation, and the behavior of the child when presented within an unfamiliar set-up, such as in the dental clinic [77]. To simplify the clinical procedure and make fissure sealant application more acceptable to young children, a shorter etching time may be used to decrease the chance of saliva contamination. As mentioned earlier, several studies showed that the length of etching time has a minimal effect on sealant retention [64]. Another measure that can be used with young children in an attempt to shorten the procedure time is to use self-etching bonding agents as an alternative to the conventional acid etching technique. Several studies have shown an insignificantly lower sealant retention rate in primary teeth when self-etching bonding agents have been used, compared to conventional acid etching [72,78]. Moreover, studies have shown that using a GI sealant may be a good interim option when salivary contamination is expected because it has a higher toleration to moisture compared to resin-based sealants [31]. However, studies on the use of GI sealants in primary teeth are very limited [79] and considerably more research is therefore needed in this area [42].

## 8. Cost-Effectiveness of Dental Sealants 

A cost-effectiveness analysis can be used to analyze the cost in relation to the outcome. In the case of sealants, we must ask how many carious lesions are prevented when dental sealants are applied [80]. Dental sealants do seem to be a cost-effective intervention; sealing permanent molars reduces the total cost by preventing the need for more expensive and invasive restorative treatment. Sealants are considered to be more cost-effective if they are used with children at a high risk of caries and with teeth surfaces susceptible to caries. It is therefore recommended that sealants should be used selectively, based on the child’s caries risk and the anatomy of the fissures [5,32,81,82]. Developing methods for targeting children at a high caries risk is therefore important to ensure the cost-effective use of sealants [82]. Perception of the susceptibility of pits and fissures to caries varies from practitioner to practitioner, when simple terms such as “deep occlusal anatomy”are used. Practitioners should be aware of the teeth and teeth surfaces that are most susceptible to caries and include them in treatment planning for sealants. For example, deep, narrow, I-shaped fissures are relatively more caries-susceptible, compared to shallow, wide, V-shaped fissures [22]. Newly erupted permanent first molars should also be seen as susceptible teeth, prior to full eruption. Dentists should think about how to protect such teeth from getting carious and whether to seal at an early or late stage of eruption. Buccal pits and lingual grooves are also considered caries-susceptible areas that are difficult to seal [83]. Sealant application is part of caries management protocol for high caries risk patients [84]. It is therefore important to evaluate to what extent other preventive approaches are used, such as professional topical fluoride application, regular daily toothbrushing with fluoridated toothpaste, the use of fluoride supplements, and diet counseling [59,84]. Caries risk is assessed using indicators such as low socio-economic status, previous caries experience, sugar consumption between meals, the presence of active white spot lesions, and low salivary flow [84].

A study showed that risk-based sealing improves clinical outcomes and saves money over never sealing. It should also be mentioned that sealing permanent molars in all patients further improves the outcome, adding only a small incremental cost relative to risk-based sealing [85]. A recent Cochrane review concluded that sealants have proved to be effective in preventing caries in high caries risk children [35]. Another study concluded that sealing primary molars reduces restorations and extractions, but is more expensive than not sealing [81]. It is therefore recommended that, to be more cost-effective, sealants be used only in children at a caries risk of develping caries [32].

## 9. Sealing Non-Cavitated Carious Lesions

Non-cavitated carious lesions refer to initial caries lesion development without any cavitation. They are defined by a change in color, surface structure, and glossiness due to demineralization before macroscopic breakdown occurs. Re-establishing the balance between remineralization and demineralization may stop the progress of caries leaving a clear clinical sign of past disease [1]. 

Due to the difficulty in diagnosing non-cavitated occlusal caries, dentists may have been inadvertently sealing caries over the years [36]. Many studies suggest that caries progression is slowed or arrested under sealants [86]. Blocking the bacterial nutritional supply may be the explanation for the arrest of caries progression observed under sealants [87]. A meta-analysis examined the caries progression under sealed permanent teeth. Six studies were included in the analysis, representing 840 teeth. Four of them sealed non-cavitated lesions and the other two used sealant over restorations. The median annual percentage of progression of non-cavitated caries lesions was 2.6% for sealed teeth and 12.6% for not-sealed teeth. This suggests that sealing non-cavitated lesions is effective in reducing progression [86]. Another randomized, controlled trial evaluated the progression of non-cavitated dentinal lesions under sealants. They included 30 molars in the sealant group (experimental group) and 30 molars in the no-sealant group (control group). The results showed a remarkable difference between the two groups; at the eight month-recall, 25 out of 26 molars (96.1%) in the control group showed caries progression. At the 12-month-recall, three out of 26 molars (11.5%) in the experimental group were present with caries progression. These molars were observed to have partial or complete sealant loss. The partial and total loss of sealant thus limited the effectiveness in arresting caries lesions [88]. Moreover, a recent critical appraisal provided evidence from clinical trials about sealing incipient occlusal caries lesions and concluded that caries lesions do not progress under well-sealed surfaces. However, the clinical success of sealing non-cavitated lesions is dependent on the complete retention of the sealants [89]. 

Sealing non-cavitated carious lesions seems to also have an effect on bacterial count. A systematic review that included six studies reported that sealing was associated with at least a 10-fold decrease in bacterial counts. About 47% percent of sealed teeth had viable bacteria, compared to 89% of unsealed lesions. They concluded that sealants were effective in reducing bacterial counts in carious lesions, but a limited number of organisms neverthless persisted [87]. Another recent systematic review supported sealing non-cavitated dentinal lesions and concluded that resin-based sealants are able to arrest the caries progression of non-cavitated dentinal lesions, while GI sealants showed low retention rates and are not able to arrest caries progression [90].

Dentists, on the other hand, have not yet adopted these findings in their clinical decision-making. A questionnaire was mailed to a randomly selected sample of 2400 dentists, of whom 771 responded. When there was no radiographic evidence of caries extending to dentin, only 38.2% of the dentists claimed that they would seal the tooth’s occlusal surface, and 23% chose the option of opening the fissure [91]. 

The available evidence and the recommendations from the ADA Council, as well as the AAPD guidelines, support sealing occlusal non-cavitated early carious lesions in children and young adults. However, sealants are most effective if they are regularly monitored and repaired [28,32,58].

## 10. Follow-Up (Recall-and-Repair)

The average sealant loss from permanent molars is between five to ten percent per year [83]. Regular sealant maintenance is therefore essential to maximize efficiency, maintain marginal integrity, and provide the protection given by optimal sealant coverage [32,92]. A study evaluated more than 8000 sealants over a period of ten years; its authors reported a sealant success rate of 85 percent after eight to ten years, due to the incorporation of an annual recall and repair program. Complete sealant retention without any need for resealing was 41 percent at ten years [93]. In another study where only a single sealant application was performed, 69 percent of the group with sealed surfaces were sound, whereas 17 percent of the group without sealants were sound. However, only 28 percent were completely retained after 15 years in the group with sealants [94]. Full retention of sealants can be checked visually, tactilely, and radiographically.

There were concerns about partially lost sealant in that it may leave sharp margins that trap food and eventually lead to caries [83]. An interesting systematic review aimed to evaluate if the risk of developing caries in previously sealed teeth with fully or partially lost sealant surpasses the risk in teeth that have never been sealed. Seven studies were included and the participants were aged between 5- and 14-year-old. It was found that the risk of caries development in previously sealed teeth after a four-year follow-up is less than or equal to that for never-sealed teeth. In other words, teeth with partial or complete sealant loss are not at a higher risk of developing caries compared to never-sealed teeth, and the relative risk (RR) ranged between 0.693 and 1.083 [95]. This does not suggest that operators should be less careful with the application technique of sealants or in the evaluation and maintainenace after placement. This suggests, however, that a child should not be forbidden to get the benefits of a sealant even if recall cannot be ensured [32,95].

## 11. Esterogenicity

Bisphenol-A (BPA) is the precursor chemical component of bisphenol-a dimethacrylate (Bis-DMA) and bisphenol-a glycidyl dimethacrylate (Bis-GMA), which are the most common monomers used in resin composite restorations and resin-based sealants. It is known for its estrogenic property with potential reproductive and developmental human toxicity [96,97]. BPA is not present in monomers as a raw material but as BPA derivatives that can sometimes be hydrolyzed and found in saliva [34].

It has been reported in a systematic review that high levels of BPA were found in saliva samples that had been collected immediately or one hour after resin-based sealant placement. High levels of BPA were also detected in urine samples [98]. However, a report by the American Dental Association and the American Academy of Pediatric Dentistry did not support the occurrence of adverse effects after sealant placement and described the BPA effect as a small transient effect [5,28].

Some studies have reported techniques, such as the immediate cleaning of the sealed surface, or the removal of the oxygen inhibition layer of the unreacted monomer, which is present on the outer layer of the sealant surface to reduce the amount of unreacted monomer. This can be done using a pumice or a rotating rubber cup [98], to reduce the potential BPA exposure. 

## 12. Conclusions

Pit and fissure sealant is an effective means of preventing pit and fissure caries in primary and permanent teeth. Dentists should therefore be encouraged to apply pit and fissure sealants in combination with other preventive measures in patients at a high risk of caries. Selection of sealant material is dependent on the patient’s age, child’s behavior, and the time of teeth eruption. Teeth that present with early non-cavitated carious lesions would also benefit from sealant application to prevent any caries progression. Sealant placement is a sensitive procedure that should be performed in a moisture-controlled environment. Maintenance is essential and the reapplication of sealants, when required, is important to maximize the effectiveness of the treatment.

## Figures and Tables

**Table 1:** Summary of data reported by NHANES ^1^ on the prevalence of caries and sealants.

Epidemiology	NHANES 1999–2004	NHANES 2011–2012
**Dental Caries Experience**		
**Total% in Primary Teeth**	42%	37%
Age group 2–5	28%	23%
Age group 6–8	51%	56%
**Total% in Permanent Teeth in Children**	21%	21%
Age group 6–8	10%	14%
Age group 9–11	31%	29%
**Total% in Permanent Teeth in Adolescents**		
Age group 12–19	59% permanent teeth	58% permanent teeth
**Untreated Dental Caries (Primary Teeth)**		
Age group 2–5	19%	10%
Age group 6–8	25.5%	20%
**Untreated Dental Caries (Permanent Teeth)**		
Age group 6–8	4%	3.3%
Age group 9–11	11%	8%
Age group 12–19	19.6	15%
**Sealant Prevalence (Had At Least One Sealed Permanent Tooth)**		
Age group 6–8	20%	31%
Age group 9–11	40%	49%
Age group 12–19	37%	43%

^1^ NHANES: national health and nutrition examination survey.

**Table 2:** Summary of evidence-based findings when comparing different sealant materials.

Sealant Materials	Summary of Findings	Level of Significance	Quality of Evidence
**GI vs. RBS**	**Sound occlusal surface:** GI reduces caries incidence by 37%	Non-significant	Very low
	**Non-cavitated occlusal carious lesion:** GI increases incidence of caries by 53%	Non-significant	
	**Risk of retention loss:** GI has a 5× greater risk of loss	Significant	
**GI vs. RMGI**	**Sound occlusal surface:** GI increases caries incidence by 41%	Non-significant	Very low
	**Risk of retention loss:** GI has a 3× greater risk of loss	Significant	
**RMGI vs. Polyacid-modified resin sealant**	**Sound occlusal surface:** RMGI reduces caries incidence by 56%	Non-significant	Very low
	**Risk of retention loss:** RMGI has an increased risk of loss of 17%	Non-significant	
**Polyacid-modified resin sealant vs. RBS**	**Sound occlusal surface:** Poly-acid modified resin sealant increases caries incidence by 1%	Non-significant	Very low
	**Risk of retention loss:** Poly-acid modified resin sealant has a decreased risk of loss of 13%	Non-significant	

**Table 3:** Historical development of dentin bonding agents.

Generation	Steps	Description	Examples
First Generation mid- 1950s and early 1960s	2-steps	Etching enamel only and adhesive application	Cervident (S.S. White, Lakewood, NJ, USA) No longer used
Second Generation Late 1970s	2-steps	Etching enamel only followed by adhesive application, slightly improved bond strength due to modifications in the coupling agent	Clearfil^TM^ Bond system F (Kuraray, Tokyo, Japan) Scotchbond^TM^ (3M ESPE, Saint Paul, MN, USA) Bondlite (Kerr, Orange, CA, USA) No longer used
Third Generation 1980s	3-steps	Partial removal of smear layer acid etching, primer, then unfilled adhesive resin application	Scotchbond^TM^ 2 (3M ESPE, Saint Paul, MN, USA) Clearfil^TM^ New Bond (Kuraray, Tokyo, Japan)
Fourth Generation 1990s	3-step etch-and-rinse adhesive	Complete removal of the smear layer and the formation of a hybrid layer Total etch technique (etching enamel and dentin, rinsing, primer, adhesive)	Scotchbond^TM^ Multi-Purpose (3M ESPE, Saint Paul, MN, USA) All-Bond 2^®^ (BISCO, Schaumburg, IL, USA)
Fifth Generation mid 1990s	2-step etch-and-rinse adhesive	Separate etching step, rinsing enamel and dentin, followed by application of combined primer-adhesive solution	OptiBond^®^ Solo (Kerr, Orange, CA, USA) Adper^TM^ Single Bond (3M ESPE, Saint Paul, MN, USA) Prime&Bond^®^ (DENTSPLY, York, PA, USA)
Sixth Generation late 1990s and early 2000s	2-step self-etching adhesive	Alter the smear layer forming a thin hybrid layer It is composed of an acidic primer (etchant + primer in one bottle) followed by bonding resin—no rinsing step	Clearfil^TM^ SE Bond (Kuraray, Tokyo, Japan) OptiBond^®^ Solo Plus Self-Etch (Kerr, Orange, CA, USA)
	1-step 2 component self-etching adhesive	It combines etchant, primer and adhesive in one step but requires pre-mixing before application	Adper^TM^ Prompt^TM^ L-Pop^TM^ (3M ESPE, Saint Paul, MN, USA) Xeno^®^ III (DENTSPLY, York, PA, USA)
Seventh Generation 2002	1-step self-etching adhesive	It combines etchant, primer and adhesive in a single bottle	Clearfil^TM^ S^3^ Bond (Kuraray, Tokyo, Japan) G-Bond^TM^ (GC America, Alsip, IL, USA) iBond^®^ (Heraeus Kulzer, Hanau, Germany)
Eighth Generation 2010	1-step self-etching adhesive	Acidic hydrophilic adhesive in a single bottle	Futurabond DC (Voco, Cuxhaven, Germany) Nanobonding agent
Multimode or Universal 2011	Self-etching adhesive or etch and rinse adhesive or selective enamel etching	Phosphoric acid pre-etching in total or selective etching	Scotchbond^TM^ Universal (3M ESPE, Saint Paul, MN, USA) Futurabond U (Voco, Cuxhaven, Germany)

## Abbreviations

AAPD	American Academy of Pediatric Dentistry
ADA	American Dental Association
ARBS	autopolymerizing resin based sealants
BIS GMA	bisphenol-a-glycidyl dimethacrylate
BPA	bisphenol-A
FRBS	fluoride releasing resin based sealants
FS	fissure sealant
GI	glass ionomer
LRBS	light polymerizing resin based sealants
NHANES	National health and nutrition examination survey
RBS	resin based sealants
RMGI	resin modified glass ionomer
SE	self-etch adhesives
SES	socioeconomic status
TE	total-etch concept
USA	United States of America
UV	ultraviolet
